# Effects of LPS Composition in *Escherichia coli* on Antibacterial Activity and Bacterial Uptake of Antisense Peptide-PNA Conjugates

**DOI:** 10.3389/fmicb.2022.877377

**Published:** 2022-06-20

**Authors:** Lise Goltermann, Meiqin Zhang, Anna Elisabeth Ebbensgaard, Marija Fiodorovaite, Niloofar Yavari, Anders Løbner-Olesen, Peter E. Nielsen

**Affiliations:** ^1^Department of Cellular and Molecular Medicine, Faculty of Health and Medical Sciences, Center for Peptide-Based Antibiotics, The Panum Institute, University of Copenhagen, Copenhagen, Denmark; ^2^Section for Functional Genomics, Department of Biology, University of Copenhagen, Copenhagen, Denmark

**Keywords:** peptide nucleic acid (PNA), antisense antimicrobials, bacterial uptake, cross-resistance, peptide antibiotics, lipopolysaccharide (LPS)

## Abstract

The physical and chemical properties of the outer membrane of Gram-negative bacteria including *Escherichia coli* have a significant impact on the antibacterial activity and uptake of antibiotics, including antimicrobial peptides and antisense peptide-peptide nucleic acid (PNA) conjugates. Using a defined subset of *E. coli* lipopolysaccharide (LPS) and envelope mutants, components of the LPS-core, which provide differential susceptibility toward a panel of bacterial penetrating peptide (BPP)-PNA conjugates, were identified. Deleting the outer core of the LPS and perturbing the inner core only sensitized the bacteria toward (KFF)_3_K-PNA conjugates, but not toward conjugates carrying arginine-based BPPs. Interestingly, the chemical composition of the outer LPS core as such, rather than overall hydrophobicity or surface charge, appears to determine the susceptibility to different BPP-PNA conjugates thereby clearly demonstrating the complexity and specificity of the interaction with the LPS/outer membrane. Notably, mutants with outer membrane changes conferring polymyxin resistance did not show resistance toward the BPP-PNA conjugates, thereby eliminating one possible route of resistance for these molecules. Finally, envelope weakening, through deletion of membrane proteins such as OmpA as well as some proteins previously identified as involved in cationic antimicrobial peptide uptake, did not significantly influence BPP-PNA conjugate activity.

## Introduction

Multidrug-resistant bacterial infections constitute a looming global public health crisis, in particular concerning Gram-negative bacteria. One of the general challenges of targeting Gram-negative (vs. Gram-positive) bacteria reside in the much more resilient outer membrane containing lipopolysaccharides (LPS). Antisense antimicrobial agents based on peptide nucleic acid (PNA) or phophorodiamidatemorpholino oligomers (PMOs) have shown promising properties for drug discovery as a new class of antibiotics (Good and Nielsen, 1998; Pifer and Greenberg, 2020). These compounds can be designed to circumvent most known antibiotic resistance mechanisms, exhibit high level of biological stability, and show specificity and flexibility in target choice. Typically, antibacterial antisense agents are designed to target the translation start codon/Shine-Dalgarno sequence in the mRNA of essential bacterial genes resulting in translation inhibition (Dryselius et al., 2003). However, the Gram-negative outer membrane, and in particular the LPS part, presents a significant barrier for bacterial uptake of both PNAs and PMOs as evidenced by the hypersusceptible, LPS-deficient AS19 strain (Good and Nielsen, 1998). Therefore, in order to facilitate uptake, antisense oligomers are conjugated to bacterial penetrating peptides (BPPs), which are typically cationic peptides (also including hydrophobic residues) without significant intrinsic antimicrobial activity (Good et al., 2001). BPPs are analogous to cell-penetrating peptides (CPPs) used for delivery to eukaryotic cells with some overlap in structure and function, and are also chemically related to some antimicrobial peptides, except that they (should) have only weak (if any) inherent antibacterial activity. Because of their composition, the BPP-PNA conjugates differ significantly in structure from any conventional antibiotics, and at present, their interaction with and passage of the bacterial envelope is incompletely understood (Frimodt-Møller et al., 2021; Yavari et al., 2021).

The outer membrane of Gram-negative bacteria contains LPS, anchored through a lipid A moiety, which together with Kdo (3-deoxy-D-manno-oct-2-ulosonic acid) and heptose make up the inner core of the LPS (Raetz and Whitfield, 2002). The (Kdo)_2_-lipid A complex is usually the minimal structure required to sustain growth. The inner core includes the Kdo and one or more L-glycero-D-manno-heptose (Hep) residues and is highly conserved. In contrast, the outer core exists as five different carbohydrate structures in *E. coli* (K12, R1, R2, R3, and R4). The outer core is also the anchor point for the highly variable O-antigen polysaccharide, which is a determinant of virulence and a means for serotyping strains. LPS structures containing the inner core, the outer core, and an antigen are denoted as smooth (S-LPS), while those lacking the antigen are denoted as rough (R-LPS), and those only possessing the inner core as deep-rough. In addition to the LPS structures, the outer membrane harbors a range of lipoproteins and outer membrane proteins, which help maintain the integrity of the envelope (Paradis-Bleau et al., 2014).

It has been established that bacterial tolerance to certain cationic antibacterial peptides can be achieved *via* LPS modification or through deletion of specific outer membrane proteins, but no single mechanism has been identified for resistance toward cationic peptides (Joo et al., 2016; Frimodt-Møller et al., 2021). However, some commonalities exist between phenotypes observed to be tolerant toward peptide-based antimicrobials such as PagP-mediated lipid A acylation and PmrAB-mediated addition of phosphoethanolamine (pEtN) (*via* EptA activation) and aminoarabinose (L-Ara4N) (*via* ArnT activation) to the LPS, thereby mitigating the negative charge of the LPS core (McPhee et al., 2003; Moskowitz et al., 2004; Herrera et al., 2010). Furthermore, *E. coli* isolates as well as other clinically relevant Gram-negative species represent a plethora of different LPS and antigen compositions. The aim of this study was to gain insight into the influence of the composition and properties of the Gram-negative outer membrane, on the activity and cellular uptake of antisense BPP-PNA conjugates. Previous studies have shown that variation in the bacterial cell surface alters the susceptibility toward AMPs (antimicrobial peptides) to varying degrees (Ebbensgaard et al., 2015). Therefore, we included different carrier peptides conjugated to a well-characterized PNA targeting the AUG-region of the essential *acpP* gene, involved in fatty acid synthesis and proven to be an effective antisense target for PNA (Good et al., 2001; Dryselius et al., 2003; Ghosal et al., 2013; Hansen et al., 2016; Yavari et al., 2021). The well-described and commonly used (KFF)_3_K-peptide was included as BPP along with two arginine-based and three AMP (antimicrobial peptide) derived peptides.

## Materials and Methods

### Strains, Growth Media, and PNA

All strains used in this study are listed in Supplementary Table S1. *E. coli* strains WD101 and WD101Δ*eptA*Δ*arnD* (WD101ΔΔ) were kindly provided by Professor M. Stephen Trent (University of Georgia, USA). *E. coli* strains DB wild type, DB L5, and DB L9 were kindly provided by Dr. Douglas Browning (University of Birmingham, UK). Single deletion strains for envelope mutants were obtained from the Keio-collection (Baba et al., 2006). All strains were grown in non-cation adjusted Muller Hinton broth (MHB) (Sigma, Cat. No. 70192). BPP-PNA conjugates (Table 1) were obtained as described previously (Good et al., 2001; Ghosal et al., 2013; Hansen et al., 2016).

**Table 1:** BPP-PNA conjugates (mm = mismatch) used.

**PNA**	**Peptide**	**Charge**	**RT**	**Target**
2301	–	+1	14	*acpP*
2108	H-(KFF)_3_K-eg1-	+5	26	*acpP*
3723	H-(KFF)_3_K-eg1-	+5		mm for PNA2108
3986	H-(R-X-R)_4_-X-(β-Ala)-	+9	17	*acpP*
3987	H-(R-X-R)_4_- X-(β-Ala)-	+9		mm for PNA3986
4099	H-(R-X)_6_-(β-Ala)-	+7	18	*acpP*
4483	H-(R-X)_6_-(β-Ala)-	+7		mm for PNA4099
4030	-Cys- RAGLQFPVGRVHRLLRK-NH_2_ (BF2A)	+6.5		*acpP*
4243	-Cys-RAGLQFPVGRVHRLLRK-NH_2_ (BF2A)	+6.5		mm for PNA4030
4449	-Cys-RAGLQFPVGRVHRLLRK-R-X-R-NH_2_ (BF2A)	+8.5		*acpP*
4128	-Cys-GKPRPYSPRPTSHPRPIRV-NH_2_ (Drosocin)	+6.5		*acpP*
4242	-Cys-GKPRPYSPRPTSHPRPIRV-NH_2_ (Drosocin)	+6.5		mm for PNA4128
4448	-Cys-GKPRPYSPRPTSHPRPIRV-R-X-R-NH_2_	+8.5		*acpP*
4124	-Cys-VDKPPYLPRPRPPRRIYNR-NH_2_ (oncocin)	+6		*acpP*
4700	-Cys-VDKPPYLPRPRPPRRIYNR-NH_2_ (oncocin)	+6		mm for PNA4124
5872	H-(KFF)_3_K-eg1-Cys(BODIPY)-	+4		*acpP*
5873	H-(KFF)_3_K-eg1-Cys(BODIPY)-	+4		mm for PNA5872

*The total charge of the conjugate is listed along with the HLPC retention time (RT) in minutes. The PNAs were synthesized as previously described (Hansen et al., 2016). eg1: 8-amino-3,6-dioxaoctanoic acid. X: 6-aminohexanoic acid. Cys: cysteine. BODIPY: 4,4-difluoro-4-bora-3a,4a-diaza-s-indacene. Anti acpP PNA sequence: H-CTCATACTCT-NH_2_, corresponding mismatch (mm) PNA: H-CTCTTACACT-NH_2_*.

### Minimum Inhibitory Concentration (MIC) and Minimum Eradication Concentration (MEC) Determination

Minimum inhibitory concentration (MIC) values were determined by broth microdilution according to standard protocols adapted to peptide-based antimicrobial compounds (Goltermann and Nielsen, 2020). Briefly, 190 μl bacterial cell culture containing ≈10^5^ cfu/ml was dispensed into a 96-well plate (Thermo Scientific, Nunc Cat. No. 260896, 96F straight w/lid) along with 10 μl PNA stock solution or antibiotic. The plate was incubated in a Tecan Genios plate reader at 37°C for 18 h, and OD was measured every 20 min at 595 nm. The MIC was determined as the lowest concentration, which inhibited visible growth (OD < 0.1) in the wells. For determination of minimum eradication concentration (MEC), cultures from the MIC 96-well plate were replicated onto Luria Bertani Agar (Sigma-Aldrich, L2897), incubated at 37°C for 18 h and analyzed visually. Experiments were performed in triplicates as a minimum.

### Time-Kill Curves

Samples were prepared as for the MIC-assay. Every hour for 4 h, 10 μl cell culture was removed, diluted in 0.9% NaCl, and plated on LB-agar. Survival was enumerated as cfu per ml after overnight incubation. Experiments were performed in triplicates and mean ± SD are shown. *P*-values were calculated using Student's *t*-test in GraphPad Prism 9.

### Acriflavin Agglutination Assay

Agglutination assay was adapted from Pampana (1933). Briefly, bacteria were scraped directly from an LB-agar plate into 1 ml of 0.9% NaCl or taken directly from an overnight culture and mixed with 0.5 ml of 0.2% acriflavine (Acriflavine hydrochloride, Sigma A8251). Agglutination was checked after 10–30 min incubation at room temperature. Experiments were performed a minimum of three times and one representative experiment is shown.

### Surface Charge and Hydrophobicity

A total of 1.8 ml of an overnight culture was harvested and washed twice in 0.9% NaCl (hydrophobicity) or phosphate-buffered saline (PBS) (charge).

For determination of surface hydrophobicity (Oguri et al., 2016), washed cells were resuspended in 0.9% NaCl to an OD (595 nm) of 1. A total of 1.4 ml cell suspension was added 300 μl *n*-hexadecane and vortexed for 1 min. After allowing the phases to separate for 30 min, the OD of the aqueous phase was measured. Surface hydrophobicity was calculated as the percentage of OD extracted into the *n*-hexadecane.

For cell charge determination *via* cytochrome c binding (Peschel et al., 1999), washed cells were resuspended in 20 mM 3-(N-morpholino)propanesulfonic acid (MOPS) (pH 7) to an OD of 7. A solution of 15 μl cytochrome c (0.5 mg/ml final concentration) in 20 mM MOPS (cytochrome c from equine heart, Sigma-Aldrich cat. no. C-2506) was added to 285 μl cell suspension. Samples were vortexed, left to stand for 10 min, and centrifuged for 5 min at 8,000 rpm before the absorbance of remaining cytochrome c in the supernatant was measured on 200 μl in a 96-well plate at 530 nm using a BioTek Synergy H1 microplate reader (BioTek Instruments, VT, USA). Control samples included cytochrome c incubated in MOPS without bacteria. Experiments were repeated 3–6 times and data were presented as mean ± SD.

### Flow Cytometry

*E. coli* cells were cultured in MHB overnight and diluted 500 × into fresh media and grown to exponential phase at OD_595_ = 0.2. The cells were pelleted and resuspended in PBS buffer containing 2 μM PNA5873, and then incubated for 1 h at room temperature. Gating was performed based on the profile of untreated *E. coli* MG1655 to exclude abnormal cell sizes and aggregates. The cell suspension was profiled using a CytoFLEX Flow Cytometer (Beckman Coulter, IN, US) and the data were analyzed using Flowlogic software (FlowLogic, Melbourne, Australia). Experiments were performed in triplicate. Data are presented as one representative flow cytometry profile and as mean fluorescence value ± SD for quantification. *P*-values were calculated using Student's *t*-test in GraphPad Prism 9.

### HPLC-Analysis of BPP-PNA Relative Hydrophobicity

Hydrophobicity of the BPP-PNA conjugates was measured using reversed phase high-performance liquid chromatography (HPLC) on an RP18 column (150 ×3.9 mm DeltaPak, 5 μm; Waters Corporation, Milford, MA, USA). Briefly, all the BPP-PNA conjugates were dissolved in water and mixed. The mixture of BPP-PNA conjugates has been run on HPLC and recorded at 260 nm. The HPLC buffers used in this study are buffer A composed of 5% acetonitrile, 0.1% trifluoroacetic acid (TFA), and 95% H_2_O, and buffer B composed of 95% acetonitrile, 0.1% TFA, and 5% H_2_O; the gradient starts from 100% of buffer A, 0% of buffer B, and ends with 60% of buffer A, 40% of buffer B in 30 min. Hydrophobicity of the compound was directly correlated to the HPLC retention time of each conjugate.

### LPS Extraction and Analysis

Approximately 10^9^ bacteria were harvested from overnight cultures and LPS extracted using commercial LPS extraction kit (LPS extraction kit, Fisher Scientific NC9753815). LPS was analyzed by sodium dodecyl sulfate (SDS) gel electrophoresis and stained using commercial LPS staining kit (Pro-Q™ Emerald 300 Lipopolysaccharide Gel Stain Kit, Thermo Fisher Scientific P20495). Finally, LPS was visualized by 300 nm UV-transilluminator with CCD camera using the protocol for SYPRO^®^ Ruby stain visualization. One representative gel is shown.

## Results

### The O-Antigen Has Limited Effects on BPP-PNA Activity

**Figure 1:**
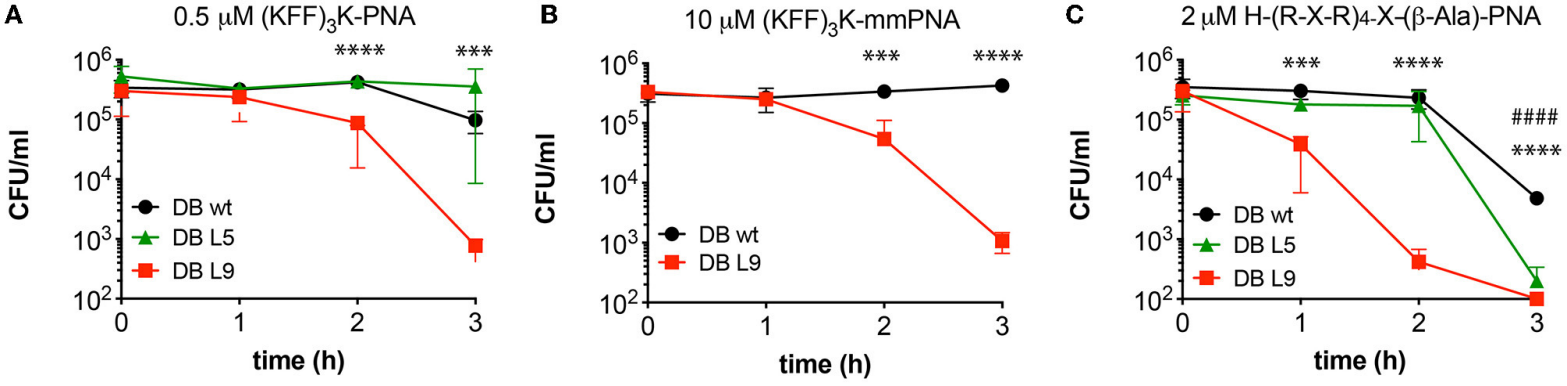
The effect of O16 antigen restoration on BPP-PNA susceptibility. The O-antigen restored strain DB L9 was compared with the O-antigen deficient MG1655 parent strain with respect to survival upon BPP-PNA treatment at concentrations bordering the MIC. Approximately 5 × 10^5^ cfu/ml were treated with BPP-PNA conjugates at the MIC value for up to 3 h. Every hour, samples were collected and plated on LB-agar to assess bacterial survival. Colonies were enumerated following overnight incubation. Time-kill curves of (KFF)_3_K-PNA match **(A)**, and (KFF)_3_K-PNA mismatch **(B)** and H-(R-X-R)_4_-X-(β-Ala)-PNA **(C)** on *E. coli* strains DB wild type (MG1655, no O-antigen), DB L5 (partly restored O-antigen), and DB L9 (O16 antigen). Experiments were performed in triplicates and data are represented as the mean ± SD. *P*-values were determined by Student's *t*-test comparing DBwt and DB L9 (^*^) or DBwt and DB L5 (#) (^***^p < 0.005, ^****^p < 0.001, ####*p* < 0.001).

The outer membrane of *E. coli* typically contains antigen appendages, and strains can be characterized and distinguished by their antigen pattern, which also has profound effects on virulence and resistance toward host defense peptides (Silhavy et al., 2010; Browning et al., 2013). To test the influence of a simple O-antigen on BPP-PNA activity, strain MG1655, which is devoid of O-antigen, and two derivatives, DB L5 (partly restored O-antigen) and DB L9 (fully restored O16-antigen) (Browning et al., 2013), were tested in a MIC and time-kill assay (Table 2, Figure 1). An acriflavin assay confirmed that MG1655 and DB L5 react as rough strains (LPS without antigen), while DB L9 displays a smooth phenotype (antigen attached to the LPS core) (Supplementary Figure S1) (Shearer and Legakis, 1985). No significant differences in MIC values were found between strains with or without O-antigen (Table 2), but the O-antigen restored DB L9 strain exhibited faster time-kill kinetics than the wild type (MG1655) for both the (KFF)_3_K- and the (R-X-R)_4_-PNA (PNA2108 and PNA3986, respectively) conjugates (Figures 1A,C), thereby indicating a relatively higher bactericidal effect. Interestingly, the mismatch control PNA (PNA3723) also exhibits faster time-kill kinetics for the DB L9 strain (Figure 1B). However, this was observed at much higher concentrations than for the fully matched PNA. This antibacterial effect is ascribed to an AMP type of mechanism of the mismatch construct. The O-antigen has previously been implicated in cationic AMP tolerance indicative of a protective role of the O-antigen maintaining the integrity of the envelope (Allen et al., 1998; Loutet et al., 2006), while we observe increased bactericidal activity in the O16-antigen restored strain compared with the rough parent strain MG1655. Thus, outer membrane appendages such as the O-antigen may act both as protectors against some compounds penetrating the envelope and as potential binding sites for other compounds that may disrupt the envelope.

**Table 2:** Minimum inhibitory concentration (μM) of BPP-PNA conjugates toward LPS mutant strains.

**Strain**	**PNA (2301)**	**(KFF)_**3**_K-PNA (2108)**	**(KFF)_**3**_K-PNA mm (3723)**	**(R-X-R)_**4**_-PNA (3986)**	**(R-X-R)_**4**_-PNA mm (3987)**	**(R-X)_**6**_-PNA (4099)**	**(R-X)_**6**_-PNA mm (4483)**	**SDS (%)**	**Colistin (μg/ml)**
K12 MG1655	*>32*	*1*	*>4*	*2*	*>8*	*1*	*8*	*0.12*	*0.03*
DB L5	*n.d*.	*0.5*	*n.d*.	*n.d*.	*n.d*.	*n.d*.	*n.d*.	*n.d*.	*n.d*.
DB L9	*n.d*.	*0.5*	*>4*	*1–2*	*n.d*.	*1*	*n.d*.	*n.d*.	*n.d*.
ATCC25922	*>16*	*1*	*>4*	*2*	*8*	*1–2*	*>8*	*0.12*	*0.06*
120 Δ*rfaC*	*n.d*.	*0.12–0.06*	*1*	*0.5–1*	*4–8*	*1*	*4*	* ≤ 0.03*	*0.03–0.016*
121 Δ*rfaE*	*n.d*.	*0.12–0.06*	*1*	*1*	*4–8*	*1*	*4*	* ≤ 0.03*	*0.03–0.016*
122 Δ*rfaF*	*>4*	*0.03*	*1*	*1–2*	*4–8*	*0.5*	*4*	* ≤ 0.03*	*0.016*
123 Δ*rfaG*	*>16*	*0.12–0.06*	*>1*	*1–2*	*8*	*0.5*	*8*	* ≤ 0.03*	* ≤ 0.016*
R1* (48 h)	*n.d*.	*0.25*	*2*	*>2*	*n.d*.	*2*	*n.d*.	*n.d*.	*0.016*
R2	*n.d*.	*1*	*>4*	*2–4*	*>8*	*4*	*>8*	*>0.5*	*0.03*
R3	*>16*	*1*	*>4*	*0.25–0.5*	*>8*	*0.25*	*4–8*	*>0.5*	*0.06*
R4	*>16*	*0.5*	*>4*	*0.25*	*8*	*0.25*	*4*	* ≤ 0.06*	*0.03*
WD101	*n.d*.	*1*	*>8*	*1*	*>16*	*1*	*>8*	*0.12*	*4*
WD101ΔΔ	*n.d*.	*0.5–1*	*>8*	*1*	*16*	*1*	*8*	*0.12*	* ≤ 0.016*
AS19	*2*	*0.12*	*1–2*	*0.5*	*2*	*0.25*	*1*	* ≤ 0.03*	*n.d*.

*X, 6-aminohexanoic acid; n.d., not determined*.

### Truncation of the LPS Inner Core Only Affects (KFF)_3_K-PNA Susceptibility

Previous studies using the LPS-impaired AS19 strain have implicated the intact outer membrane as a major barrier for uptake of BPP-PNA antisense conjugates (Good et al., 2000), in particular for compounds such as (KFF)_3_K-PNA (PNA2108) for which SbmA functions as an inner membrane transporter (Ghosal et al., 2013; Yavari et al., 2021). AS19 is indeed significantly more susceptible than strain MG1655 to the (KFF)_3_K and slightly more susceptible to the RX-type BPP-PNA conjugates (Table 2). However, although the full genome sequence of AS19 is available (Avalos et al., 2018), the LPS of AS19 has not yet been fully characterized. Thus, we decided to use a series of defined LPS inner core mutants, which have previously been used to investigate the uptake of antimicrobial peptides (Ebbensgaard et al., 2015), in order to obtain a deeper insight into the features of the LPS responsible for this effect.

**Figure 2:**
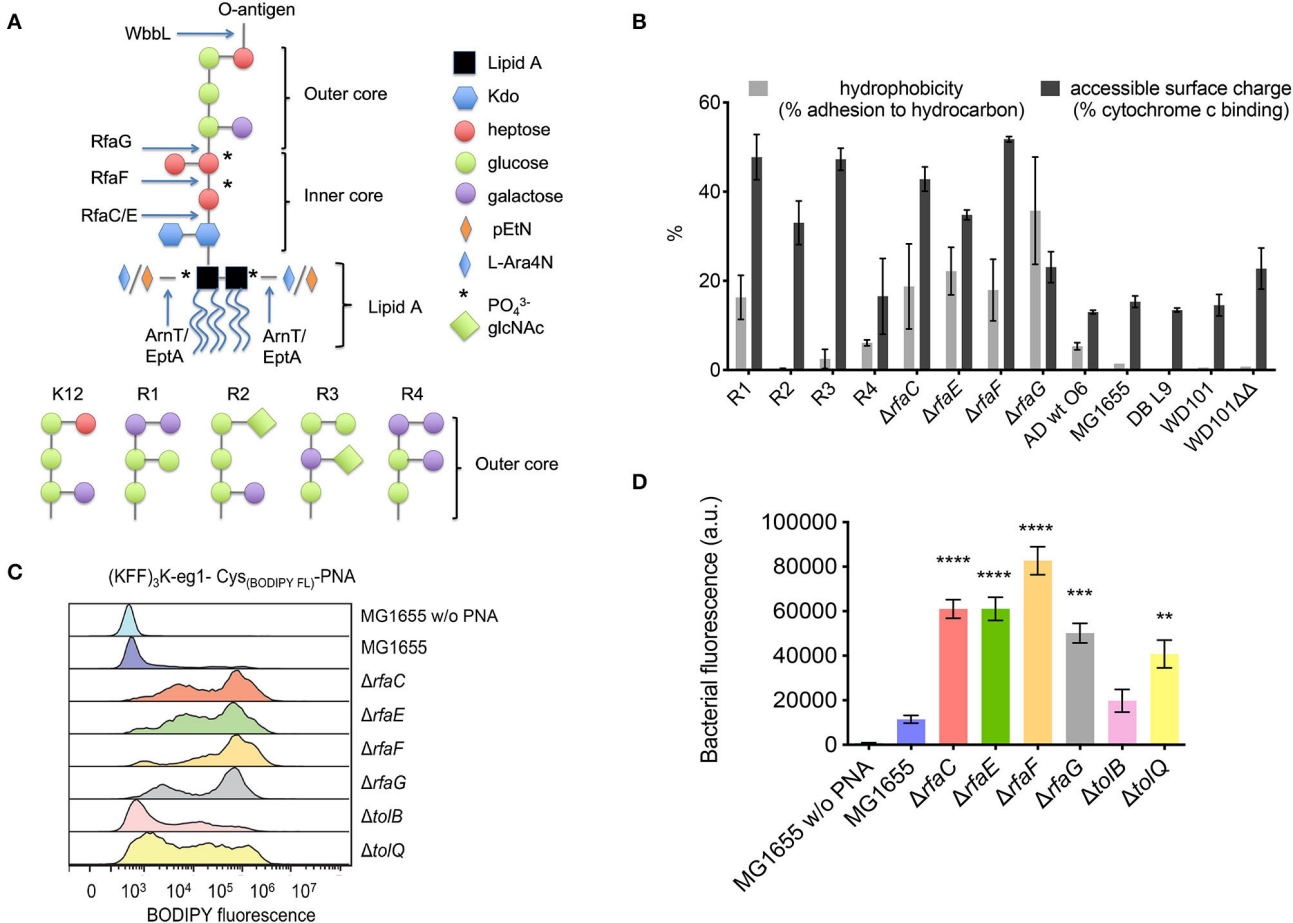
**(A)** Structure of LPS inner and outer core mutants. Arrows indicate where the LPS structure terminates in the indicated mutants. RfaC and RfaF catalyze the addition of heptose, while RfaE is required for the biosynthesis of the heptose precursor and RfaG is a glucosyltransferase attaching glucose to the inner core. **(B)** Hydrophobicity and accessible surface charge of the tested LPS mutants as measured by adherence to hydrocarbon or cytochrome c binding, respectively. **(C)** Flow cytometry histogram of uptake of BODIPY fluorophore-labeled (KFF)_3_K-PNA in *E. coli* mutant strains. The indicated strains were incubated with 2 μM fluorophore-labeled (KFF)_3_K-mmPNA (5873) for 1 h. Flow cytometry profiles were normalized and the mean bacterial fluorescence **(D)** used as a measure to compare conjugate uptake. Experiments were performed in triplicates and data are represented as the mean ± SD. Student's *t*-test was used to determine significant differences between the wild-type MG1655 and the mutant strains (^**^p < 0.01, ^***^p < 0.005, ^****^p < 0.001).

Four inner core mutants (Δ*rfaC*, Δ*rfaE*, Δ*rfaF*, and Δ*rfaG*) with increasingly shortened LPS core (Figure 2A) were investigated. The deletions span the three heptosyl and glycosyltransferases *rfaC, rfaF*, and *rfaG* responsible for attaching heptose and glucose, respectively, to the growing LPS core, and *rfaE* required for the synthesis of the heptose precursor needed for LPS synthesis (Figure 2A). The LPS profile of these strains was qualitatively characterized by LPS extraction and gel electrophoretic separation, and all showed similar profiles to the AS19 strain (Supplementary Figure S2). Furthermore, all strains were characterized with respect to hydrophobicity and accessible cell surface charge (Figure 2B, *vide infra*). All four mutants were significantly (8–32 times) more susceptible to the (KFF)_3_K-based BPP-PNA (MIC 0.03–0.12 μM) than the parent strain (MIC 1 μM), and interestingly, the susceptibility was also increased toward the mismatch control (Table 2). It is known that the peptide portion of the conjugates can have a membrane disruptive effect at high concentrations (Eriksson et al., 2002). This effect is most likely exacerbated in mutants lacking significant parts of the LPS, and would indicate analogous molecular mechanism for the BPP membrane translocation activity and the inherent (albeit much weaker) antibacterial activity of the (KFF)_3_K peptide as well as all of the (RX)_6_ and (RXR)_4_ peptides. The Δ*rfaF*-mutant showed the greatest reduction in MIC. The reason for this is not known, but the same trend was observed for Cecropin P1 and Cecropin B, although these peptides are in general less affected by deletions to the inner core (Ebbensgaard et al., 2018).

Interestingly, the four inner core mutants showed much smaller differences in susceptibility to the arginine-based BPP-PNAs (Table 2), supporting that the outer membrane is not the major rate limiting barrier for the uptake of arginine-rich BPP-PNAs (Frimodt-Møller et al., 2021). All the inner core mutants as well as AS19 are also significantly more susceptible to SDS (MIC < 0.03%) than the parent strain (MIC 0.12%), which should indicate a general destabilization of the outer membrane (Table 2).

If the higher BPP-PNA susceptibility of *E. coli* with compromised LPS structure is due to easier penetration of the envelope, a correlation between antibacterial activity and bacterial uptake should exist. Therefore, the uptake of fluorophore-labeled BPP-PNA was measured by flow cytometry (Figures 2C,D). We used the (KFF)_3_K-PNA, as only this conjugate showed significant differences in MIC toward the LPS mutants. The mismatch (KFF)_3_K-PNA conjugate (PNA5873) was used for flow cytometry studies to avoid bactericidal effects while measuring the uptake of the conjugate.

Although addition of a fluorophore generally (slightly) increases the MIC (unpublished results), the data confirm that uptake of the conjugate is increased in mutants with lower MICs compared with the wild-type strain MG1655 (Figure 2C). The inner core LPS mutants clearly contain a higher fraction of the labeled conjugate compared with the wild type as evidenced by the higher mean fluorescence measured for the bacterial cell population (Figure 2D). Cellular uptake was confirmed by confocal laser scanning microscopy to rule out surface attachment (unpublished results). The flow cytometry profiles (Figure 2C) indicate a complex distribution, which is not understood at present, but similar population heterogeneity of uptake at low concentrations of AMPs has previously been reported (Pérez-Peinado et al., 2018).

The data support that the outer LPS layer is a very significant part of the outer membrane major barrier for uptake and thus for antibacterial activity of (KFF)_3_K-PNA conjugates, which are translocated across the inner membrane by the SbmA transporter (Ghosal et al., 2013), and consequently, it follows that perturbation of the LPS should greatly increase access to this transporter, hence increasing susceptibility.

Removing the outer LPS core causes an increase in the overall accessible negative charge of the bacteria as measured by increased adhesion to positively charged cytochrome c in a binding assay (Figure 2B), presumably because of the increased exposure of phosphate groups in the LPS inner core. Also, hydrophobicity increased as measured by increased binding to hexadecane (Figure 2B) using the BATH (bacterial adhesion to hydrocarbons) assay. Both properties would be expected to increase the affinity of the cationic, partly hydrophobic BPP-PNA-conjugates (Supplementary Figure S3) to the bacterial cell surface. However, no direct correlation between BPP-PNA susceptibility and cell surface charge or hydrophobicity for the different strains is apparent. Presumably, the overall structure and chemical composition of the LPS is of greater importance.

### Different Core Types Display Different BPP-PNA Susceptibilities

The LPS-core composition of *E. coli* varies between the K-12-type and four other core types (R1–R4, Figure 2A) differing in the arrangement of sugar moieties that make up the outer LPS core. These differences in turn result in variation of the hydrophobicity and accessible surface charge (Figure 2B).

Surprisingly, the R1 core type exhibited slow growth in non-cation adjusted MHB compared with LB, and the MIC assay was therefore conducted over 48 h. Despite this growth defect, the R1 core type showed approximately the same tolerance toward the R-X-R-PNAs as the K-12 strain (MIC 2 μM), while being slightly more susceptible to the (KFF)_3_K-PNA (MIC 0.25 μM vs. 1 μM) (Table 2).

The R2 core type was the only tested strain with slightly increased tolerance toward the R-X-R-PNAs (in particular PNA4483, for which the MIC increased from 1 to 4 μM), while the effect of the (KFF)_3_K-PNA was similar to the K-12 strain. The R3 and R4 core types were about four times more susceptible to the arginine-based BPP-PNAs than other core types, and these were the only of all the tested strains with increased susceptibility toward the R-X-R-PNAs (Table 2).

For AMPs, the two core types R3 and R4 do not display similar susceptibilities. For instance, the R4 core type is significantly more susceptible to melittin than the R3 core type (Ebbensgaard et al., 2018).

The hyper-permeable AS19 is most often characterized by its high susceptibility toward SDS, which is interpreted as a result of a “leaky” LPS phenotype. Interestingly, we also found that the R4 core type displays increased susceptibility toward SDS compared with MG1655, while the opposite is the case for the R3 and R2 core type (Table 2). This further underlines the fact that even seemingly small alterations in the LPS composition, which are not necessarily accompanied by measurable changes in overall hydrophobicity or charge, can change the susceptibility toward BPP-PNA as well as detergents and AMPs.

### Conjugates Containing Naturally Occurring AMPs

Peptide nucleic acid can also be delivered into bacteria by some non-lytic naturally occurring AMPs (Hansen et al., 2016). We tested three of these AMP carriers (buforin, drosocin, and oncocin) for their activity toward the Δ*rfaC* mutant to establish if the LPS constituted the main barrier for uptake of this type of BPP-PNAs (Table 3).

**Table 3:** MIC values (μM) of BPP-PNA constructs composed of naturally occurring BPPs; buforin 2a (Buf), drosocin (Dro), and oncosin (Onc) conjugated to the anti-*acpP*-PNA.

**Strain**	**Buf-PNA (4030)**	**Buf-PNA mm (4243)**	**Buf-R-X-R-PNA (4449)**	**Buf**	**Dro-PNA (4128)**	**Dro-PNA mm (4242)**	**Dro-R-X-R-PNA (4448)**	**Dro**	**Onc-PNA (4124)**	**Onc-PNA mm (4700)**
ATCC25922	0.5	>8	1	32	1	>8	2	8–16	>2	>8
ATCC25922 Δ*rfaC*	<0.25	<2	<0.25	32	<0.06	<1	<0.12	2–4	0.25	4
MG1655	1	n.d.	<0.5	32	0.5–1	n.d.	1–2	>32	8	n.d.
MG1655 Δ*sbmA*	8–16	n.d.	>4	16	4–8	n.d.	2	>32	>8	n.d.

*R, arginine; X, 6-aminohexanoic acid*.

PNA4128 and its corresponding mismatch control PNA4242 contain drosocin as the BPP conjugated to the anti-*acpP*-PNA. This construct was found to be SbmA-dependent (Hansen et al., 2016) and would therefore be expected to have characteristics similar to those of the (KFF)_3_K-conjugates. Indeed, the MIC for the drosocin-PNA-conjugate (PNA4128) is more than 16-fold lower in the in Δ*rfaC*-mutant (MIC < 0.06 μM) than the ATCC25922 control (1 μM). The drosocin peptide alone is non-lytic (Gobbo et al., 2002) and seemingly SbmA-independent [(Hansen et al., 2016), Table 3]. However, the susceptibility toward drosocin is also increased in the Δ*rfaC*-mutant (2–4 μM vs. 8–16 μM) probably because the LPS is a significant barrier to prevent unconjugated drosocin from reaching its intracellular target. Furthermore, the buforin (PNA4030) and oncocin (PNA4124) PNA-conjugates are SbmA dependent and more active against the Δ*rfaC*-mutant, although this is not the case for buforin alone (Table 3). Thus, the properties of the carrier peptide are not necessarily fully mirrored in the corresponding PNA conjugate. Finally, attachment of an R-X-R-group to the BPP-PNA conjugate (PNA4448) changed the drosocin-conjugate from SbmA dependent to SbmA independent, while the buforin-R-X-R BPP-PNA (PNA4449) remained SbmA dependent (Hansen et al., 2016), further highlighting the detailed complexity of peptide carrier design (Table 3).

### Polymyxin Resistance Does Not Result in BPP-PNA Resistance

Lack of resistance development or existing resistance and cross-resistance in clinically important strains is a prerequisite for the success of any novel antibiotic drug discovery. Resistance toward cationic antimicrobial peptides has been tied to modification of the LPS layer. Specifically, polymyxin resistance can be obtained through activation of *pmrA*, in turn activating *arnT* and *eptA*, which catalyze the addition of L-4-aminoarabinose (L-Ara4N) and phosphoethanolamine (pEtN), respectively, to the phosphate groups on lipid A. The outcome of this is a change in the overall surface charge and increased tolerance toward polymyxin (Herrera et al., 2010). In order to test for cross-resistance to the BPP-PNAs, the polymyxin-resistant *pmrA*^C^ strain (WD101) and the re-sensitized *pmrA*^C^Δ*arnT*Δe*ptA* (WD101ΔΔ) strain were used (Table 2). No significant difference in MIC was found between these two strains as well as control strains for any of the tested BPP-PNA conjugates (Table 2), suggesting lack of cross-resistance between polymyxin and the BPP-PNA conjugates.

### Bacterial Envelope Perturbation Offers Only Limited Increase in BPP-PNA Uptake

Perturbation of the bacterial cell envelope by deletion of bacterial envelope proteins could be expected to destabilize the outer membrane to such an extent as to allow the entry of BPP-PNAs. The Keio collection has repeatedly been screened to identify knock-out mutants with altered susceptibility or permeability toward conventional antibiotic or small molecules (Liu et al., 2010; Paradis-Bleau et al., 2014). A screen to identify strains with selectively altered susceptibility toward BPP-PNA conjugates only yielded one significant result, which was the identification of the SbmA transporter as being involved in the transport of (KFF)_3_K-PNA conjugates across the inner membrane, whereas no clones were identified for the arginine-based BPP-PNAs (Ghosal et al., 2013).

We re-examined this phenomenon by selecting a range of strains, which have previously been identified as either permeable because of cell envelope disruptions (Paradis-Bleau et al., 2014) or responsible for the entry of conventional antibiotics (Liu et al., 2010) or AMPs (Lazzaroni et al., 2002) into *E. coli*, and these were tested against the BPP-PNAs (Table 4). SurA, a periplasmic chaperone, and EnvC have both been implicated in AMP resistance (Justice et al., 2005; Oguri et al., 2016) as well as identified as contributing to envelope integrity (Paradis-Bleau et al., 2014). However, while the *surA* mutant showed increased susceptibility toward the BPP-PNAs (2–8 fold reductions in the MIC), this was not the case for the *envC* mutant. Other *E. coli* mutants, which have been identified as having increased permeability such as *gpmI, hydN, metL, mrcB, ompA*, and *ppiB*, did not seem to allow increased uptake of any of the BPP-PNAs, maybe because of the large size of these conjugates (~5 kDa).

**Table 4:** MIC values of permeable *E. coli* BW25113 mutants from the Keio collection (8).

**Strain**	**(KFF)_**3**_K-PNA (2108) (μM)**	**H-(R-X)_**6**_-PNA (4099) (μM)**	**H-(R-X-R)_**4**_-X-PNA (3986) (μM)**	**Carbenicillin (μg/ml)**
MG1655	0.5–1	1–2	1–2	16
BW25113	1	1	1	8
**envC**	0.5–1	1	0.5	8
**gpmI**	0.5	0.5–1	0.5	8
hydN	0.5	2	2	16
metL	0.5	2	2	16
**mrcB**	0.5	1–2	1	4
ompA	1	1	n.d.	8
**ppiB**	0.5	1–2	1–2	16
**surA**	0.125	0.5	0.5	4
**pal**	0.5	n.d.	n.d.	n.d.
tolA	0.5	2	0.5	16
tolB	0.125	0.5	1	≤ 2
**tolQ**	0.125	0.5	0.5	≤ 2
**tolR**	0.125	0.5	0.5	2–4
**ompT**	0.5	n.d.	1	n.d.
**ycaC**	1	n.d.	0.5–1	n.d.

*Mutants marked in bold were identified as antibiotic susceptible in (24)*.

*X, 6-aminohexanoic acid*.

The Pal-Tol-system, an envelope spanning complex involved in maintaining the outer membrane integrity, has previously been implicated in general antibiotic susceptibility, and thus was also investigated. Interestingly, only *tolR* and *tolQ* mutants were identified as being especially susceptible to antibiotics in a screen of the Keio-collection (Liu et al., 2010), while in a different screen, each component of this system *pal, tolB* and *tolQ, tolR, and tolA* showed increased susceptibility to a range of antibiotics, albeit in a different strain background (Kowata et al., 2016). The *tolB, tolQ*, and *tolR* mutants only showed significantly increased susceptibility toward (KFF)_3_K-PNA but not toward R-X-R-PNA (Table 4). This suggests that the integrity of the outer membrane is significantly compromised in these strains as further evidence by the circa 8-fold increase in carbenicillin susceptibility (Table 4).

We also addressed the uptake in the *tolB* and *tolQ* mutants through flow cytometry. As evident from the histograms (Figure 2C), the uptake of the fluorescently labeled (KFF)_3_K-PNA was indeed increased in both mutants compared with the wild type. Most likely, the effect of any of the envelope mutations will greatly depend on the culture conditions. This would explain why phenotypes vary for similar mutants throughout the literature. Furthermore, the composition of the membranes can change in response to alterations in the protein composition. For example, it has been demonstrated that *tolB*-mutants have an increased number of OmpC porins in the membrane (Lazzaroni, 1986). We also included two mutants, namely, *ompT* and *ycaC*, which have been associated with tolerance toward the cationic peptides protamine and apideacin 1B, respectively (Stumpe et al., 1998; Schmidt et al., 2016). Neither mutant showed any difference in susceptibility toward the BPP-PNAs compared with the wild type, thus ruling out cross-resistance (Table 4). The *ompT* mutant has also been tested against a panel of other antimicrobial peptides of natural origin and showed no changes in the MIC for any of these (Ebbensgaard et al., 2018).

## Discussion

A range of LPS and outer membrane mutants of *E. coli* was investigated in order to explore the influence of the composition of the Gram-negative outer membrane with the efficacy of BPP-PNA antisense antimicrobials.

The presence of the O-antigen results in a slight increase in the rate of bacterial killing by the BPP-PNAs possibly because these cationic conjugates bind to the O-antigen. However, this effect was marginal and did not influence the MIC.

While some AMPs, such as polymyxin, eradicate bacteria by destabilizing the outer and inner membrane, the peptide part of the BPP-PNA-conjugates is only a means to carry the PNA over the membranes and should ideally not possess any intrinsic antibacterial activity. In this sense, the peptides may be expected to behave more like proline-rich AMPs, which also have intracellular targets and show limited membrane damage. However, many of these are SbmA-dependent, and while this is also true for the (KFF)_3_K-PNAs, the R-X-R based peptides are in general SbmA-independent and no transporter has yet been identified for the R-X-R based BPP-PNAs (Ghosal et al., 2013). Instead, it seems that several genetic changes resulting in a decreased potential over the inner membrane can increase tolerance toward arginine-based BPP-PNA conjugates (Frimodt-Møller et al., 2021). Thus, uptake and activity of SbmA-transported (KFF)_3_K-PNA is primarily limited by translocation across the LPS/outer membrane, whereas uptake and activity of the SbmA-independent RXR-PNA type is predominantly limited by inner membrane translocation. This, in part, explains the effect observed for the LPS inner-core mutants. Perturbation of the LPS inner core allows easier access to the inner membrane and the SbmA transporter for the (KFF)_3_K-PNAs, while other BPP-PNAs may less easily translocate across the inner membrane making this rate limiting and thus diminishing the effect of removing the LPS barrier. Indeed, the SbmA dependency may predominantly be due to degradation of the peptide prior to reaching the inner membrane as observed for the (KFF)_3_K-type BPP-PNAs (Yavari et al., 2021).

Although the different outer core-type strains varied significantly in their response to the different classes of BPP-PNA conjugates, none of these variations were directly correlated with alterations in cell surface physicochemical properties, such as hydrophobicity or charge. Specifically, no direct correlation between the measured accessible surface charge and the tolerance toward the different BPP-PNA conjugates was clear. In contrast, it has been reported that an alteration of the overall bacterial cell surface charge is responsible for polymyxin resistance in *E. coli* and *Salmonella* species. However, the mechanisms, which lead to polymyxin resistance in the WD101 strain, do not convey resistance toward any of the tested conjugates, although they are all cationic. Additionally, no resistance mechanism has yet been discovered, which provides cross-resistance to multiple AMPs in general through alterations of the LPS. On the contrary, major as well as minor changes to the chemical composition of the LPS can dramatically change the susceptibility toward peptide-based antimicrobials. We have identified only a few gene deletions (*surA, tolB, Q*, and *R*), which destabilized the bacterial cell envelope sufficiently to allow generally increased passage of the tested BPP-PNA conjugates. The effect was only significant for the (KFF)_3_K-PNA, which is known to be excluded primarily by the outer membrane (in SbmA^+^ strains). Furthermore, single gene deletions, which have been implicated in the susceptibility toward other AMPs, had no effect on the BPP-PNA conjugates.

## Conclusion

The results of this study demonstrate that the composition of the Gram-negative outer membrane among a range of *E. coli* strains influences the susceptibility toward BPP-PNA conjugates depending on the delivery peptide and thus the mode of envelope translocation. Thus, the carrier peptide portion of the conjugates may be optimized based on the target strains. This is a unique feature of these compounds, which in combination with optimization of the gene-sequence target can guide the development of species/strain-selective precision antibiotics and may also allow rapid adjustment of the conjugates in case of resistance development through outer membrane adaptation. Importantly, we identified no cross-resistance with other classes of antimicrobial compounds in strains where outer membrane alterations cause reduced susceptibility toward these. Therefore, design, selection, and characterization of carrier peptides should also consider the outer membrane composition of the target strain.

## Data Availability Statement

The original contributions presented in the study are included in the article/Supplementary Material, further inquiries can be directed to the corresponding author/s.

## Author Contributions

LG, AE, AL-O, and PN designed the experiments. LG, MZ, MF, and NY performed the experiments. LG and PN wrote the manuscript. All authors contributed to the article and approved the submitted version.

## Funding

This work was supported by the NovoNordisk Foundation Challenge Program (NNF16OC0021700).

## Conflict of Interest

The authors declare that the research was conducted in the absence of any commercial or financial relationships that could be construed as a potential conflict of interest.

## Publisher's Note

All claims expressed in this article are solely those of the authors and do not necessarily represent those of their affiliated organizations, or those of the publisher, the editors and the reviewers. Any product that may be evaluated in this article, or claim that may be made by its manufacturer, is not guaranteed or endorsed by the publisher.
