# Supplementary material for: Effects of LPS Composition in Escherichia coli on Antibacterial Activity and Bacterial Uptake of Antisense Peptide-PNA Conjugates
Source: Front Microbiol. 2022 Jun 20;13:877377. doi: 10.3389/fmicb.2022.877377 (PMC9251361; doi:10.3389/fmicb.2022.877377)
Supplement: Supplementary file 1 [file Data_Sheet_1.docx]

Supplementary Material

# Supplementary Figures and Tables

**
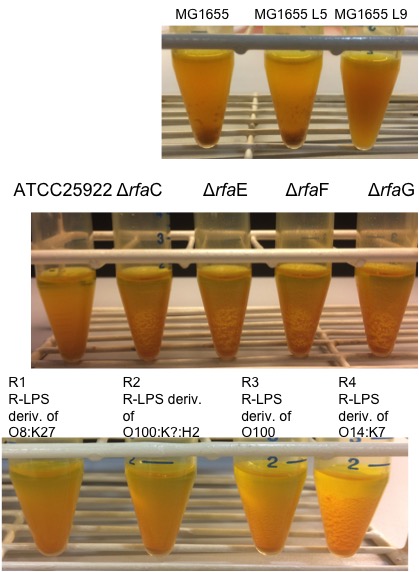
**

**Figure S1. Acriflavin agglutination assay. Agglutination in acriflavin confirms the rough surface of bacterial strains. MG1655 with the restored O-antigen *wbb* locus (L9) displays a smooth phenotype. All *rfa* mutants showed a strong agglutination phenotype as did the rough mutants of the 4 different core types (R1-4).**

**
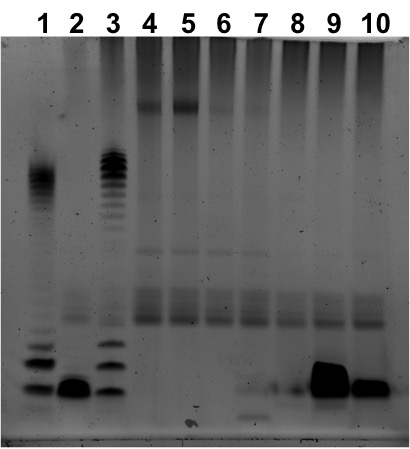
**

**Figure S2. SDS-PAGE gel of purified LPS stained using the Pro-Q Emerald 300 glycoprotein stain. 1) Smooth *E.coli* LPS standard, 2) MG1655, 3) ATCC25922, 4) ATCC25922Δ*rfaC*, 5) ATCC25922Δ*rfaE*, 6) ATCC25922Δ*rfaF*, 7) ATCC25922Δ*rfaG*, 8) AS19, 9) WD101, 10) WD101Δ*eptA*Δ*arnT***

**
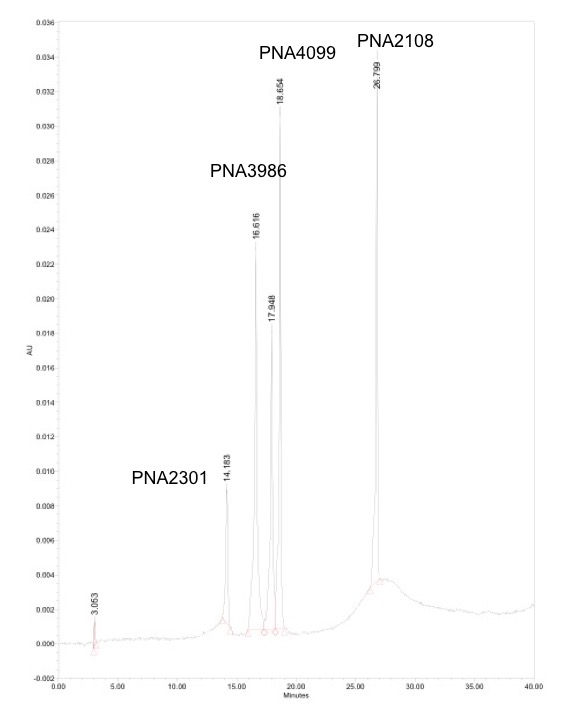
**

**Figure S3. HPLC of the peptide-PNAs used throughout this study. Longer retention times are a result of higher hydrophobicity.**

| **strain** | **genotype** | **phenotype** | **reference** |
| --- | --- | --- | --- |
| MG1655 | *F-,*λ*-, rph-1* | Wild-type | Lab collection |
| MG1655Δ*sbmA* | *F-,*λ*-, rph-1,* Δ*sbmA* | Increased tolerance to (KFF)_3_K-conjugated PNAs | (Ghosal et al., 2013) |
| DB L5 | MG1655 with *wbbL* integrated into the *rfb* gene cluster but retaining original IS*5* insertion | Partially restored O-antigen biosynthesis | (Browning et al., 2013) |
| DB L9 | MG1655 with *wbbL* integrated into the *rfb* gene cluster | Fully restored O-antigen biosynthesis | (Browning et al., 2013) |
| AS19 |  | hyperpermeable | (Sekiguchi and Iida, 1967) |
| ATCC25922  (ADwt) | Clinical isolate, Serotype O6, Biotype 1 | Wild-type | ATCC strain collection |
| AD120 | ATCC25922 Δ*rfaC* | Perturbed inner LPS core, increased sensitivity towards certain CAMPs | (Ebbensgaard et al., 2015) |
| AD121 | ATCC25922 Δ*rfaE* | Perturbed inner LPS core, increased sensitivity towards certain CAMPs | (Ebbensgaard et al., 2015) |
| AD122 | ATCC25922 Δ*rfaF* | Perturbed inner LPS core, increased sensitivity towards certain CAMPs | (Ebbensgaard et al., 2015) |
| AD123 | ATCC25922 Δ*rfaG* | No outer LPS core, increased sensitivity towards certain CAMPs | (Ebbensgaard et al., 2015) |
| *E.coli* F470 (R1) | *E. coli* R1 prototype; R-LPS derivative of O8:K27 |  | (Schmidt et al., 1969; Vinogradov et al., 1999) |
| *E.coli* F632 (R2) | *E. coli* R2 prototype; R-LPS derivative of O100:K? (B):H2 |  | (Hämmerling et al., 1971; Heinrichs et al., 1998) |
| *E.coli* F653 (R3) | *E. coli* R3 prototype; R-LPS derivative of O100 |  | (Schmidt et al., 1970) |
| *E.coli* F2513 (R4) | *E. coli* R4 prototype; R-LPS derivative of O14:K7 | Increased sensitivity towards certain CAMPs | (Schmidt et al., 1974; Ebbensgaard et al., 2018) |
| BW25113 | F-, Δ(*araD-araB*)567, Δ*lacZ*4687(::rrnB-3), λ-, *rph*-1, Δ(*rhaD-rhaB*)568, *rhdR*514 | Wild-type | (Baba et al., 2006)  Keio collection parent |
| Δ**envC** | Δ*envC*725::kan | Increased susceptibility to LL-37 | (Baba et al., 2006; Oguri et al., 2016) |
| Δ**gpmI** | Δ*pgm736*::kan | Increased OM permeability | (Baba et al., 2006; Paradis-Bleau et al., 2014) |
| Δ**hydN** | Δ*hydN*788::kan | Increased OM permeability | (Baba et al., 2006; Paradis-Bleau et al., 2014) |
| Δ**metL** | Δ*metL727*::kan | Increased OM permeability | (Baba et al., 2006; Paradis-Bleau et al., 2014) |
| Δ**mrcB** | Δ*mrcB765*::kan | Increased OM permeability | (Baba et al., 2006; Paradis-Bleau et al., 2014) |
| Δ**ompA** | Δ*ompA772*::kan | Increased OM permeability | (Baba et al., 2006; Paradis-Bleau et al., 2014) |
| Δ**ppiB** | Δ*ppiB737*::kan | Increased OM permeability | (Baba et al., 2006; Paradis-Bleau et al., 2014) |
| Δ**surA** | Δ*surA765*::kan | Increased antimicrobial susceptibility | (Justice et al., 2005)  (Baba et al., 2006) |
| Δ**pal** | Δ*pal790*::kan | Increased antimicrobial susceptibility | (Baba et al., 2006; Kowata et al., 2016) |
| Δ**tolA** | Δ*tolA788*::kan | Increased antimicrobial susceptibility | (Baba et al., 2006; Kowata et al., 2016) |
| Δ**tolB** | Δ*tolB789*::kan | Increased antimicrobial susceptibility | (Baba et al., 2006; Kowata et al., 2016) |
| Δ**tolQ** | Δ*tolQ786*::kan | Increased antimicrobial susceptibility | (Baba et al., 2006; Kowata et al., 2016) |
| Δ**tolR** | Δ*tolR787*::kan | Increased antimicrobial susceptibility | (Baba et al., 2006; Kowata et al., 2016) |
| Δ**ompT** | Δ*ompT774*::kan | Hypersusceptible to protamine | (Stumpe et al., 1998; Baba et al., 2006) |
| Δ**ycaC** | Δ*ycaC721*::kan | Apideacin 1b tolerant | (Baba et al., 2006; Schmidt et al., 2016) |
| WD101 | W3110 constitutive pmrA mutant, PmbR | Colistin tolerant | (Herrera et al., 2010) |
| WDΔeptA  ΔarnT | WD101, ∆eptA::nptII, ∆arnT::cat, KanR , CamR | Colistin sensitive | (Herrera et al., 2010) |

**Table S1. Strains used in the study.**

**References**

Baba, T., Ara, T., Hasegawa, M., Takai, Y., Okumura, Y., Baba, M., et al. (2006). Construction of Escherichia coli K-12 in-frame, single-gene knockout mutants: the Keio collection. *Mol. Syst. Biol.* 2, 2006.0008. doi:10.1038/msb4100050.

Browning, D. F., Wells, T. J., França, F. L. S., Morris, F. C., Sevastsyanovich, Y. R., Bryant, J. A., et al. (2013). Laboratory adapted Escherichia coli K-12 becomes a pathogen of Caenorhabditis elegans upon restoration of O antigen biosynthesis. *Mol. Microbiol.* 87, 939–950. doi:10.1111/mmi.12144.

Ebbensgaard, A., Mordhorst, H., Overgaard, M. T., Aarestrup, F. M., and Hansen, E. B. (2018). Dissection of the antimicrobial and hemolytic activity of Cap18: Generation of Cap18 derivatives with enhanced specificity. *PLoS ONE* 13, e0197742. doi:10.1371/journal.pone.0197742.

Ebbensgaard, A., Mordhorst, H., Overgaard, M. T., Nielsen, C. G., Aarestrup, F. M., and Hansen, E. B. (2015). Comparative Evaluation of the Antimicrobial Activity of Different Antimicrobial Peptides against a Range of Pathogenic Bacteria. *PLoS ONE* 10, e0144611. doi:10.1371/journal.pone.0144611.

Ghosal, A., Vitali, A., Stach, J. E. M., and Nielsen, P. E. (2013). Role of SbmA in the uptake of peptide nucleic acid (PNA)-peptide conjugates in E. coli. *ACS Chem. Biol.* 8, 360–367. doi:10.1021/cb300434e.

Hämmerling, G., Lüderitz, O., Westphal, O., and Mäkelä, P. H. (1971). Structural investigations on the core polysaccharide of Escherichia coli 0100. *Eur. J. Biochem.* 22, 331–344. doi:10.1111/j.1432-1033.1971.tb01549.x.

Heinrichs, D. E., Monteiro, M. A., Perry, M. B., and Whitfield, C. (1998). The assembly system for the lipopolysaccharide R2 core-type of Escherichia coli is a hybrid of those found in Escherichia coli K-12 and Salmonella enterica. Structure and function of the R2 WaaK and WaaL homologs. *J. Biol. Chem.* 273, 8849–8859. doi:10.1074/jbc.273.15.8849.

Herrera, C. M., Hankins, J. V., and Trent, M. S. (2010). Activation of PmrA inhibits LpxT-dependent phosphorylation of lipid A promoting resistance to antimicrobial peptides. *Mol. Microbiol.* 76, 1444–1460. doi:10.1111/j.1365-2958.2010.07150.x.

Kowata, H., Tochigi, S., Kusano, T., and Kojima, S. (2016). Quantitative measurement of the outer membrane permeability in Escherichia coli lpp and tol-pal mutants defines the significance of Tol-Pal function for maintaining drug resistance. *J. Antibiot.* 69, 863–870. doi:10.1038/ja.2016.50.

Oguri, T., Yeo, W.-S., Bae, T., and Lee, H. (2016). Identification of EnvC and Its Cognate Amidases as Novel Determinants of Intrinsic Resistance to Cationic Antimicrobial Peptides. *Antimicrob. Agents Chemother.* 60, 2222–2231. doi:10.1128/aac.02699-15.

Paradis-Bleau, C., Kritikos, G., Orlova, K., Typas, A., and Bernhardt, T. G. (2014). A genome-wide screen for bacterial envelope biogenesis mutants identifies a novel factor involved in cell wall precursor metabolism. *PLoS Genet.* 10, e1004056. doi:10.1371/journal.pgen.1004056.

Schmidt, G., Fromme, I., and Mayer, H. (1970). Immunochemical studies on core lipopolysaccharides of Enterobacteriaceae of different genera. *Eur. J. Biochem.* 14, 357–366. doi:10.1111/j.1432-1033.1970.tb00297.x.

Schmidt, G., Jann, B., and Jann, K. (1969). Immunochemistry of R lipopolysaccharides of Escherichia coli. Different core regions in the lipopolysaccharides of O group 8. *Eur. J. Biochem.* 10, 501–510. doi:10.1111/j.1432-1033.1969.tb00717.x.

Schmidt, G., Jann, B., and Jann, K. (1974). Genetic and immunochemical studies on Escherichia coli O14:K7:H-. *Eur. J. Biochem.* 42, 303–309. doi:10.1111/j.1432-1033.1974.tb03340.x.

Schmidt, R., Krizsan, A., Volke, D., Knappe, D., and Hoffmann, R. (2016). Identification of New Resistance Mechanisms in Escherichia coli against Apidaecin 1b Using Quantitative Gel- and LC-MS-Based Proteomics. *J. Proteome Res.* 15, 2607–2617. doi:10.1021/acs.jproteome.6b00169.

Sekiguchi, M., and Iida, S. (1967). Mutants of Escherichia coli permeable to actinomycin. *Proc. Natl. Acad. Sci. U.S.A.* 58, 2315–2320. Available at: http://pubmed.gov/4173585.

Stumpe, S., Schmid, R., Stephens, D. L., Georgiou, G., and Bakker, E. P. (1998). Identification of OmpT as the protease that hydrolyzes the antimicrobial peptide protamine before it enters growing cells of Escherichia coli. *J. Bacteriol.* 180, 4002–4006. Available at: http://eutils.ncbi.nlm.nih.gov/entrez/eutils/elink.fcgi?dbfrom=pubmed&id=9683502&retmode=ref&cmd=prlinks.

Vinogradov, E. V., Drift, K. V. D., Thomas-Oates, J. E., Meshkov, S., Brade, H., and Holst, O. (1999). The structures of the carbohydrate backbones of the lipopolysaccharides from Escherichia coli rough mutants F470 (R1 core type) and F576 (R2 core type). *Eur. J. Biochem.* 261, 629–639. doi:10.1046/j.1432-1327.1999.00280.x.
